# Ultra-Stable and Durable Piezoelectric Nanogenerator with All-Weather Service Capability Based on N Doped 4H-SiC Nanohole Arrays

**DOI:** 10.1007/s40820-021-00779-0

**Published:** 2021-12-13

**Authors:** Linlin Zhou, Laipan Zhu, Tao Yang, Xinmei Hou, Zhengtao Du, Sheng Cao, Hailong Wang, Kuo-Chih Chou, Zhong Lin Wang

**Affiliations:** 1 Beijing Advanced Innovation Center for Materials Genome Engineering, Collaborative Innovation Center of Steel Technology, University of Science and Technology Beijing, Beijing, 100083 People’s Republic of China; 2 Beijing Institute of Nanoenergy and Nanosystems, Chinese Academy of Sciences, Beijing, 100083 People’s Republic of China; 3 MOE Key Laboratory of New Processing Technology for Non-Ferrous Metals and Materials, Guangxi Key Laboratory of Processing for Non-Ferrous Metals and Featured Materials, Guangxi University, Nanning, 530004 People’s Republic of China; 4 School of Materials Science Engineering, Zhengzhou University, Zhengzhou, 450001 People’s Republic of China

**Keywords:** Piezoelectric nanogenerators, N doped 4H-SiC nanohole arrays, Environmental actuation sources, All-weather service capability, Enhanced short circuit current density

## Abstract

Ultra-stable piezoelectric nanogenerator (PENG) driven by environmental actuation sources with all-weather service capability is highly desirable. Here, the PENG based on N doped 4H-SiC nanohole arrays (NHAs) is proposed to harvest ambient energy under low/high temperature and relative humidity (RH) conditions. Finite element method simulation of N doped 4H-SiC NHAs in compression mode is developed to evaluate the relationship between nanohole diameter and piezoelectric performance. The density of short circuit current of the assembled PENG reaches 313 nA cm^−2^, which is 1.57 times the output of PENG based on N doped 4H-SiC nanowire arrays. The enhancement can be attributed to the existence of nanohole sidewalls in NHAs. All-weather service capability of the PENG is verified after being treated at -80/80 ℃ and 0%/100% RH for 50 days. The PENG is promising to be widely used in practice worldwide to harvest biomechanical energy and mechanical energy.

**Supplementary Information:**

The online version contains supplementary material available at 10.1007/s40820-021-00779-0.

## Highlights

- An ultra-stable all-weather service piezoelectric nanogenerator (PENG) with a wide operating temperature range (-80~80  ℃) and a wide operating relative humidity range (0~100%) is proposed.
- The PENG based on N doped 4H-SiC exhibits long-term service stability up to 50 days.
- The short circuit current density of PENG based on N doped 4H-SiC is enhanced significantly.

**Supplementary Information:**

The online version contains supplementary material available at 10.1007/s40820-021-00779-0.

## Graphical abstract

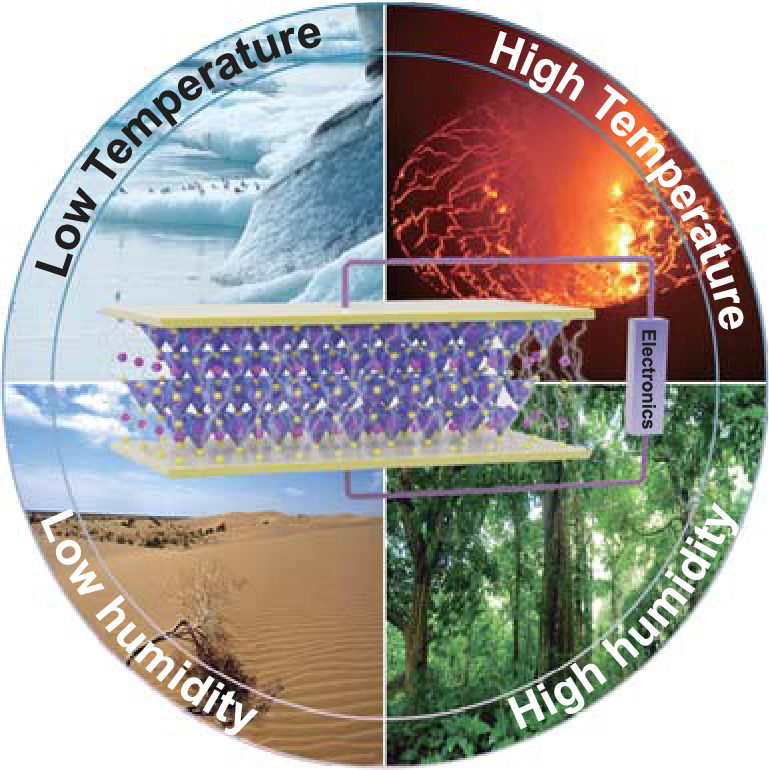

## Introduction

Environmental actuation sources, one of the most widely distributed energy sources in nature, are being explored and developed. Nowadays, nanogenerators (NGs) have been considered favorable candidates for ambient sources harvesting [1–5]. Among them, piezoelectric nanogenerator (PENG) has a tighter connection structure, smaller size and longer service life, making it more suitable for practical applications. Due to the complexity of the Earth's environment, such as from − 70 °C in polar region to 60 °C in Africa, from 0% relative humidity (RH) in desert to 100% RH in rainforest, the PENG with all-weather service capability is in urgent need of development. So, the standards required for piezoelectric materials used to assemble all-weather service PENG, such as chemical and thermal stability, environmental friendliness and durability, have become the main obstacles restricting their wide application [6]. The piezoelectric polymer, for example, PVDF, P(VDF-TrFE) and PVDF-HFP, can’t be applied in extreme temperature environments due to thermal instability [7–10]. As for piezoelectric ceramics, i.e., Pb(Zr, Ti)O_3_ (PZT), Pb(Mg_1/3_Nb_2/3_)O_3_-PbTiO_3_ (PMN-PT), CsPbBr_3_, with high piezoelectric coefficients usually contain lead (Pb), which is harmful to the environment [11–14]. While the lead-free piezoelectric ceramics, i.e., BaTiO_3_, NaNbO_3_ usually have complex preparation processes and harsh synthesis conditions, which makes them not suitable for promotion in practical applications [15–18]. In particular, the high brittleness of ceramics makes them easily damaged, limiting their service life severely. In addition to the classic piezoelectric materials, multiple piezoelectric semiconductors such as ZnO [19, 20], GaN [21, 22], MoS_2_ [23], and MoSe_2_ [24], have been widely investigated to assemble PENG. Among them, the chemical instability of ZnO to acids and bases prevents it from harvesting ambient sources. The preparation process of highly oriented GaN nanoarrays is cumbersome. And the fabrication of uniform monolayer MoS_2_ and MoSe_2_ is pretty complex to control bonding and crystal, making it impractical for global promotion [25].

SiC, one of the most important third-generation semiconductors with extraordinary chemical and thermal stability, outstanding mechanical properties and good thermal shock resistance, is recognized as one of the potential materials for constructing devices with excellent stability and durability to service in harsh conditions including high temperature, high pressure, high irradiation and high power [26–29]. Recently, our group has noted the excellent piezoelectric properties of 4H-SiC due to the separation of positive and negative charge centers along c-axis and proposed a PENG based on N doped 4H-SiC nanowire arrays (NWAs) [30, 31]. The stable output under high temperature and high concentration of acid/alkali solutions environments verifies the stability of the PENG based on 4H-SiC. However, in order to obtain the PENG with all-weather service capability, the output performance of the PENG based on 4H-SiC needs to be further improved, the stability (including different temperature and RH) and durability of the PENG need to be further explored.

Herein, an ultra-stable and durable PENG with all-weather service capability and improved output ability was fabricated by N doped 4H-SiC NHAs. The influence of nanohole diameter on structural stability and output ability of the NHAs was studied by finite element method (FEM). Especially, all-weather service capability of the PENG, including high/low temperature and RH, was investigated systematically. The results of practical applications show that N doped 4H-SiC NHAs is one of the most favorable candidates for PENG worked in harsh conditions.

## Experimental Section

### Materials

The N doped single-crystalline 4H-SiC wafer was obtained from TankeBlue Semiconductor Co. Ltd.. Ethanol (C_2_H_5_OH, 99%) and hydrogen peroxide (H_2_O_2_, 30%) were purchased from Sinopharm Chemical Reagent. Hydrofluoric acid (HF, 40%) was from Aladdin in Beijing of China.

### Materials Preparation and Fabrication of PENG

The N doped 4H-SiC NHAs were prepared by anodic oxidation. The etching solution is composed of HF, C_2_H_5_OH and H_2_O_2_ with a volume ratio of 6:6:1. The voltage of 21 V with a cycle time (T) of 8 ms and a pause time (*T*_off_) of 4 ms was applied for 10 min to form NHAs. The freestanding NHAs film was exfoliated under the function of the direct voltage of 21 V for 60 s and utilized to assemble a well-sealed PENG. PDMS was spin-coated on one side of NHAs and cured at 80 °C for 20 min and a piece of Al foil was attached to PDMS tightly. The obtained N doped 4H-SiC/PDMS/Al was fixed on another piece of Al foil by silver paste. The sandwich-structure device was fixed on a PET plate and encapsulated with PDMS.

### Characterization and Measurement

The morphology and structure of the NHAs were investigated by field emission scanning electron microscope (FESEM; JSM-6701F, JEOL) and transmission electron microscopy (TEM; JEM-2100, JEOL). The crystal structure of samples was studied by X-ray diffraction (XRD; SmartLab/Ultima IV, Rigaku). X-ray photoelectron spectroscopy (XPS; ESCALAB 250Xi, Thermo Fisher Scientific) was used to measure their surface species. Displacement-voltage butterfly loop of NWAs was recorded by the piezoresponse force microscopy (PFM; Dimension Icon, Bruker).

### Simulation Settings

The bottom of the NHAs is treated as grounding and fixing constraints. The top of NHAs with floating potential was rigid and the normal displacement of the side walls was set to zero [32]. And the pressure of 1 MPa was applied on the top surface of NHAs along the c-axis.

## Results and Discussion

**Fig. 1:**
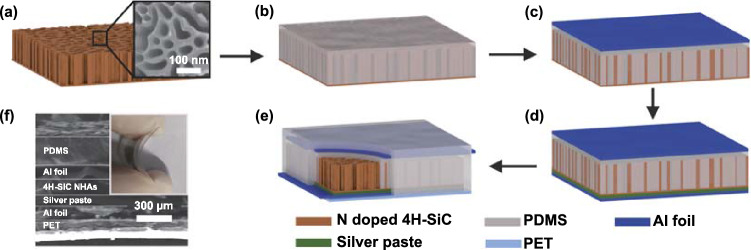
**a–e** Schematic diagram of the fabrication process for the PENG. The inset in **a** is the SEM image of the top-view of N doped 4H-SiC NHAs. **f** SEM image of the cross-sectional view of the assembled PENG. The inset in the upper right corner is the picture of bending PENG

The N doped 4H-SiC NHAs were prepared by anodic oxidation of single-crystalline N doped 4H-SiC wafer [33, 34]. The representative fabrication procedure of PENG based on the exfoliated N doped 4H-SiC NHAs is schematically illustrated in Fig. 1a-e. The SEM image of the N doped 4H-SiC NHAs in the inset of Fig. 1a reveals the actual nanohole distribution in the arrays. The cross-sectional SEM image reveals the interlayer structure of the PENG (Fig. 1f). And the excellent flexibility of the well-sealed PENG is disclosed in the inset of Fig. 1f.

### Characterization of NHAs

At the beginning of anodic oxidation, extremely small holes appear. HF etching solution tends to enter the bottom of these holes under the electric field perpendicular to the surface of the N doped 4H-SiC sheet. The holes expand gradually and CO_2_ gas generated by the oxidation of SiC accumulates on sidewalls, hindering the lateral etching reaction. Yet the longitudinal etching process proceeds normally, forming neatly arranged NHAs perpendicular to the SiC substrates. The larger pore is generated by the corrosion and penetration of the sidewalls between small holes [34]. SEM images show that the N doped 4H-SiC NHAs exhibits a dense nanohole structure with diameters ranging from 14.5 nm to more than 200 nm (Fig. S1a, b). The average diameter of nanohole in arrays is 73.67 nm and most of the apertures are less than 100 nm (Fig. S1c) [35]. The XRD patterns of the NHAs powder confirm that it can be indexed to 4H-SiC (JCPDS Card No. 73–1664) (Fig. S2a). There is only one sharp peak of (004) in the XRD spectrum of N doped 4H-SiC NHAs, disclosing their single-crystalline nature and high crystallinity. Further, the detailed morphology of the as-prepared sample was disclosed by TEM (Fig. S2b). The N doped 4H-SiC NHAs exhibits different widths at different locations, which results from the combined effects of the voltage oscillations, the periodical etching reaction and the different etching rates of the C and Si faces [34, 36]. The well-arranged crystalline lattice fringes represented in high-resolution TEM (HRTEM) image correspond to the (004) crystal plane of 4H-SiC (Fig. S2c). The select area electron diffraction (SAED) patterns (Fig. S2d) are consistent with XRD results. XPS spectrum of the NHAs reveals that they are composed of C, Si, O, and N (Fig. S3). The O 1 s spectrum indicates the presence of SiO_x_C_y_ and SiO_2_ formed during anodizing (Fig. S3d) [26]. And the N dopants incorporated into 4H-SiC lattice are revealed by N 1 s fine XPS spectrum (Fig. S3e).

Further, the displacement-voltage butterfly loops of 4H-SiC and N doped 4H-SiC verify that the N doped 4H-SiC exhibits more significant piezoelectric properties (Fig. S4). Actually, 4H-SiC belongs to hexagonal *P6*_*3*_*mc* space-group symmetry with a wurtzite structure. The tetrahedral unit of 4H-SiC is composed of one Si atom and four C atoms coordinated with the Si atom. In this unit, the apical bond length of Si–C is 1.890 Å (parallel to the c-axis) yet the basal one is 1.880 Å. The distortion of the tetrahedron along c-axis leads to the separation of the cation and anion centers of 4H-SiC, forming c-axis-oriented dipole moments. Once an external force is applied along the c-axis, the deformation of the tetrahedral units of 4H-SiC will significantly strengthen the dipole moments and enhance piezoelectricity [31, 37–39]. Furthermore, N doping will enhance the piezoelectricity of 4H-SiC by adjusting crystal structure and inducing dipoles. On one hand, the lattice distortion caused by the introduction of N atoms in 4H-SiC lattice increases the asymmetry of the wurtzite structure [40]. On the other hand, the remaining electrons of N atoms tend to become free electrons. The electron-losing N ion makes the surrounding positively charged center shift, forming a dipole. These dipoles will emerge orientation polarization under the force field, resulting in an enhanced piezoelectric effect [41]. Hence, the N doped 4H-SiC possesses more significant piezoelectricity.

### FEM Simulation of NHAs

**Fig. 2:**
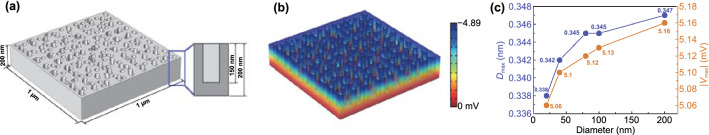
**a** Geometry Schematics of the N doped 4H-SiC NHAs. **b** Distribution of the *V* in NHAs at a pressure of 1 MPa. **c** The tendency of *D*_max_ and |*V*_max_| with the increase of nanohole diameters

To further investigate the piezoelectric effect of the N doped 4H-SiC NHAs, a finite element method (FEM) simulation was performed by the COMSOL Multiphysics software [32, 42, 43]. The geometry schematic of N doped 4H-SiC NHAs with the size of 1 μm × 1 μm × 200 nm is presented in Fig. 2a. The aperture of nanoholes in NHAs was set to vary from 40 to 200 nm according to the statistical distribution of nanoholes (Fig. S1c). The electric potential (*V*) of the N doped 4H-SiC NHAs is uniformly distributed (Fig. 2b) and the peak value of *V* (*V*_max_) reaches -4.89 mV. To distinguish the contribution of nanoholes with different apertures to performance, five NHAs units with diameters of 20, 40, 80, 100, and 200 nm were established (Fig. S5). The tendency of maximum displacement (*D*_max_) and |*V*_max_| of NHAs with increasing diameters are represented in Fig. 2c. When the same pressure is applied, the displacement and electric potential increase nonlinearly with the enlargement of nanohole diameters. Although the larger displacement of NHAs will induce better electrical output performance, the resulting giant structural deformation will greatly limit their service life in practice. Especially, the trend of electric potential and deformation growth slows down as the aperture increases. As for the as-prepared N doped 4H-SiC NHAs, the smaller holes will improve the structural stability and the larger holes can optimize the piezoelectric performance. Therefore, the NHAs with a diameter within the range of 20 to 200 nm approximately are suitable for assembling PENG. As the anodizing time increases, the nanohole size expands and the sidewalls of the holes in NHAs gradually collapse to form NWAs. Hence, the structural stability of the 4H-SiC NWAs is inevitably worse than that of the NHAs, making them inapplicable in practice.

### Performance Test of PENG

**Fig. 3:**
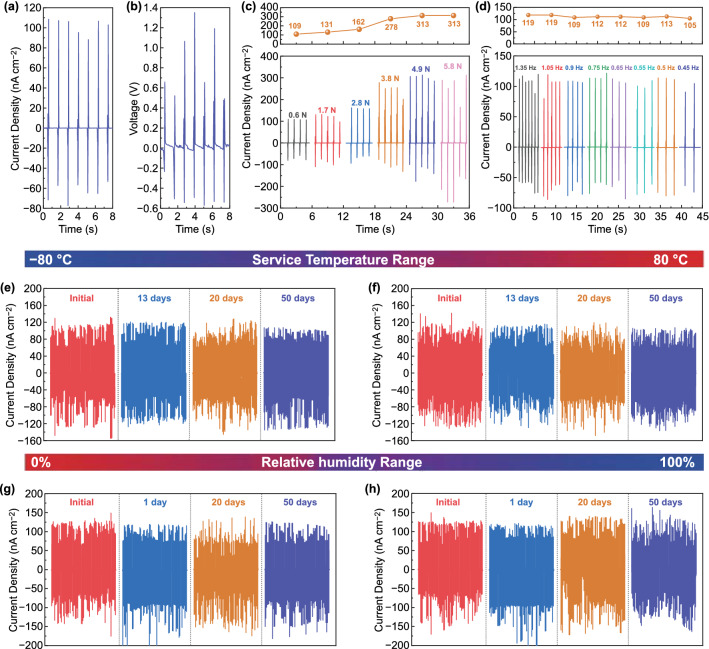
**a** Density of *I*_sc_ and **b** *V*_oc_ of the PENG. The density of *I*_sc_ and the output trend of PENG based on N doped 4H-SiC NHAs under different external stimulus: **c** various forces and **d** various frequencies. The long-term stability of as-constructed PENG within up to 50 days under different temperatures and RHs: **e** -80, **f** 80 °C, **g** 0% RH, and **h** 100% RH

When a force of 0.6 N is applied, the density of *I*_sc_ and open circuit voltage (*V*_oc_) of the assembled PENG are 108 nA cm^−2^ and 1.35 V, respectively (Fig. 3a, b). A blank PENG without N doped 4H-SiC NHAs was constructed to verify the effective piezoelectric output of NHAs. Compared with the PENG based on N doped 4H-SiC NHAs, the blank one shows a negligible signal generated by the noise in surrounding environments (Fig. S6). Hence, it can be concluded that the electrical signals originate from the piezoelectric effect of N doped 4H-SiC NHAs.

The performance of PENG based on N doped 4H-SiC NHAs under actual working conditions was evaluated by changing the force and frequency of the external stimulus. The density of *I*_sc_ rises from 108 to 313 nA cm^−2^ with external force increasing from 0.6 to 4.9 N. And then, the current remains constant as the force increases (Fig. 3c). When the force is less than 4.9 N, the deformation of the NHAs possibly increases with the increase of the force, resulting in an enhanced polarization and larger output. Once the force reaches 4.9 N or above, it might be difficult for NHAs to produce greater deformation and stronger polarization. Thus the forward output will no longer improve with the increase of force but stabilize at a fixed value [34]. It should be noted that subsequent tests are all carried out under the external force of 0.6 N. The PENG shows excellent stability under frequency interference in the range of 0.45 to 1.35 Hz (Fig. 3d). As frequency increases, the density of *I*_sc_ of the PENG fluctuates slightly within the range of 105 to 119 nA cm^−2^. The changing rate of dipole moments in NHAs is not affected by frequency fluctuations of external stimuli. So that both the escaped charges and the accumulated charges on surface remain unchanged, resulting in a stable output [44]. The insensitivity to frequency allows the as-prepared PENG to be applied in situations with multiple interference factors, such as heavy rains and typhoons.

Further, the PENG is verified to be capable of working normally after being frozen at − 80 °C (Fig. 3e) and heated at 80 °C for 50 days (Fig. 3f). There is no significant drop during the imparting and releasing processes throughout the long-term stability test, indicating the ultra-stability and durability of the PENG. Hence, the PENG can be utilized as an energy harvester to collect and transfer irregular environmental actuation sources in our living environment for a long period. In addition, The PENG also shows a stable output within the RH range of 0 to 100% for 50 days (Fig. 3g, h). As a result, the ultra-stable and durable PENG with all-weather service capability based on N doped 4H-SiC NHAs is proven to be applicable worldwide.

The load capacity of PENG was monitored by measuring *V*_oc_ across the resistor connected to the PENG. The peak value of *V*_oc_ increases nonlinearly from 0.014 to 0.574 V with the external load ranging from 1 to 100 MΩ (Fig. S7a, b). And the power density of PENG rises with the resistances increase from 1 to 30 MΩ and then decreases once the external load exceeds 30 MΩ. The NHAs-based PENG exhibits a maximum power density value of 26.52 nW cm^−2^ when the resistance of 30 MΩ is connected. Notably, the electrical energy converted from mechanical energy by PENG can be stored in capacitors through a bridge rectifier. The pulsed electrical signals are converted into forward voltage by a rectifier circuit (Fig. S7c). A 100 μF capacitor is charged to 0.033 V by the PENG within 800 s (Fig. S7d), proving the feasibility of the PENG to be applied in practical applications.

### Environmental Energy Harvesting

Here, the PENG was used to harvest biomechanical energy, i.e. finger tapping, foot striking and mechanical energy, i.e., cantilever beam, simulated automobile exhaust emission. When the PENG is subjected to finger tapping and foot striking, the density of *I*_sc_ reaches 45 and 318 nA cm^−2^, respectively (Fig. S8a, b). The mechanical energy generated from the vibration of the simulated cantilever beam can be converted to electrical energy by the PENG (55 nA cm^−2^, Fig. S8c). Besides, the automobile exhaust emission process was simulated by air blower and the PENG was used to harvest the wind and vibration energy simultaneously (− 116 nA cm^−2^, Fig. S8d). The thermal stability of N doped 4H-SiC breaks through the limitation of high temperature conditions, making the N doped 4H-SiC NHAs-based PENG can be applied to harvest multiple energy sources during the automobile exhaust emission process.

The key performance of N doped 4H-SiC NHAs-based PENG is compared with PENGs constructed by various material systems, i.e., lead-based perovskite, lead-free perovskite, piezoelectric polymer and piezoelectric semiconductor (Table 1). Notably, the density of *I*_sc_ of the PENG based on N doped 4H-SiC NHAs is basically the same as that of some PENGs assembled by classic piezoelectric materials, such as CsPbBr_3_/P(VDF-TrFE) [45], BiFeO_3_ [46], ZnO [20] and GaN [21, 22]. Most importantly, PENG based on N doped 4H-SiC NHAs possesses a wider service temperature range (− 80 ~ 80 °C), wider operating RH range (0 ~ 100%) and longer service life (50 days), indicating the all-weather service capability. In addition, the N doped 4H-SiC has been verified to be capable of working at 200 °C in our previous work [30]. The wide service temperature and RH range of the N doped 4H-SiC is of great significance to the practical application of PENG.

**Table 1:** The key performance of PENGs based on various materials

Materials		Mode	*I*_sc_	Service temperature (℃)	Relative humidity	Stable service time	Refs
Lead-based perovskite	PZT	Bending	10.9 μA cm^−2^	RT	Air	50,000 cycles	[47]
	PZT	Pressing	17.5 μA	RT	Air	–	[48]
	PMN-PT	Pressing	290 μA cm^−2^	RT	Air	–	[12]
	CsPbBr_3_/P(VDF-TrFE)	Pressing	0.17 μA	RT	Air	–	[45]
Lead-free perovskite	BaTiO_3_	Pressing	2.9 μA	RT	Air	14 days	[49]
	BiFeO_3_	Pressing	~ 250 nA	RT	Air	1000 cycles	[46]
	NaNbO_3_	Pressing	16 nA cm^−2^	RT	Air	30 h	[17]
Piezoelectric polymer	PVDF	Pressing	> 0.7 μA	RT	Air	–	[7]
	P(VDF-TrFE)/GeSe	Pressing	1.14 μA	RT	Air	–	[8]
	P(VDF-HFP)	Pressing	0.9 μA cm^−2^	RT	Air	–	[9]
Piezoelectric semiconductor	ZnO	Pressing	7.2 μA cm^−2^	RT	Air	–	[19]
	ZnO	Pressing	36 nA	RT	Air	–	[20]
	ZnO/AlN	Pressing	1.10 μA	RT	Air	–	[50]
	GaN	Bending	85.6 nA	RT	Air	20,000 cycles	[21]
	GaN	Pressing	150 nA	RT	Air	–	[22]
	AlN	Bending	1.6 μA	RT	Air	1800 cycles	[51]
	MoS_2_	Bending	–	RT	Air	~ 175 s	[23]
	MoSe_2_	Bending	–	RT	Air	> 1500 s	[24]
	N doped 4H-SiC NWAs	Pressing	200 nA cm^**−2**^	25 ~ 200	Air	20,000 cycles	[30] Our previous work
	N doped 4H-SiC NHAs	Pressing	313 nA cm^**−2**^	− 80 ~ 80 (200)	0 ~ 100%	50 days	This work

Materials-types of materials used to assemble PENGs; Mode-the working mode of the PENG, mainly including pressing and bending; *I*_sc_-short circuit current of the PENG; Service temperature-the temperature range in which the PENG works normally; Relative humidity-the humidity range in which the PENG works normally; Stable service time-the service life of the PENG in normal operation; Refs-corresponding references

The ultra-stability and enhanced performance of the PENG based on N doped 4H-SiC NHAs can be attributed to the following points. Firstly, the intrinsic properties of N doped 4H-SiC. The wide band gap, stable physical/chemical properties and intrinsic piezoelectricity of N doped 4H-SiC enable it to adapt to various extreme environments. Secondly, the nanostructure of the as-prepared NHAs. On one hand, the NHAs composed of nanoholes with different diameters exhibit both excellent structural stability and significant electrical output. On the other hand, the NHAs undergo anodic oxidation in a short period and retain the sidewalls. There are more SiC units in NHAs to produce dipoles in crystals when subjected to external stimuli, resulting in an enhanced macroscopic current output. Finally, the well-sealed structure of the PENG. The as-prepared PENG is completely wrapped to isolate external pollutions and prevent the structure from being damaged.

## Conclusions

In summary, an ultra-stable PENG based on N doped 4H-SiC NHAs with all-weather service ability is demonstrated. The assembled PENG shows the density of *I*_sc_ and *V*_oc_ of 108 nA cm^−2^ and 1.35 V when subjected to an external force of 0.6 N. Once a force of 4.9 N is applied, it produces the density of *I*_sc_ of 313 nA cm^−2^, which is 1.57 times the output of that assembled by NWAs (200 nA cm^−2^). The FEM simulation results reveal that the deformation and the electric potential of the NHAs both increase with the enlargement of the aperture. And the PENG based on NHAs with diameters ranging from 20 to 200 nm approximately possess excellent structural stability and enhanced short circuit current density. The PENG can effectively resist the interference caused by frequency varying from 0.45 to 1.35 Hz. And the PENG maintains high output after being treated at − 80/80 ℃ and 0%/100% RH for 50 days. It realizes the conversion from mechanical energy to electricity by harvesting ambient energy generated by finger tapping, foot striking, cantilever beam and simulated automobile exhaust emission. The ultra-stable and durable PENG based on the N doped 4H-SiC NHAs can harvest environmental actuation sources effectively and is of great significance for the development of self-powered systems.

## Supplementary Information

Below is the link to the electronic supplementary material.

Supplementary file1 (PDF 755 kb)
