# Supplementary material for: Ultra-Stable and Durable Piezoelectric Nanogenerator with All-Weather Service Capability Based on N Doped 4H-SiC Nanohole Arrays
Source: Nanomicro Lett. 2021 Dec 13;14:30. doi: 10.1007/s40820-021-00779-0 (PMC8669063; doi:10.1007/s40820-021-00779-0)
Supplement: Supplementary file 1 — Supplementary file1 (PDF 755 kb) [file 40820_2021_779_MOESM1_ESM.pdf]

Supporting Information for

Ultra-Stable and Durable Piezoelectric Nanogenerator with All-Weather Service Capability based on N-Doped 4H-SiC Nanohole Arrays

Linlin Zhou[1], Laipan Zhu[2], Tao Yang[1, *], Xinmei Hou[1, *], Zhengtao Du[3], Sheng Cao[3], Hailong Wang[4], Kuo-Chih Chou[1], Zhong Lin Wang[2]

[1]Beijing Advanced Innovation Center for Materials Genome Engineering, Collaborative Innovation Center of Steel Technology, University of Science and Technology Beijing, Beijing 100083, P. R. China

[2]Beijing Institute of Nanoenergy and Nanosystems, Chinese Academy of Sciences, Beijing 100083, P. R. China

[3]MOE Key Laboratory of New Processing Technology for Non-ferrous Metals and Materials, Guangxi Key Laboratory of Processing for Non-ferrous Metals and Featured Materials, Guangxi University, Nanning 530004, P. R. China

[4]School of Materials Science Engineering, Zhengzhou University, Zhengzhou 450001, P. R. China

*Corresponding authors. E-mail: yangtaoustb@ustb.edu.cn (Tao Yang), houxinmeiustb@ustb.edu.cn (Xinmei Hou)

Supplementary Figures

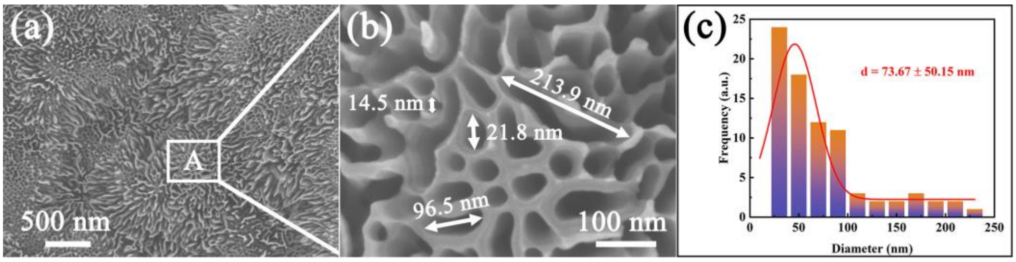

Fig. S1 (**a**, **b**) SEM images of NHAs. (**c**) The diameter distribution of N doped 4H-SiC NHAs

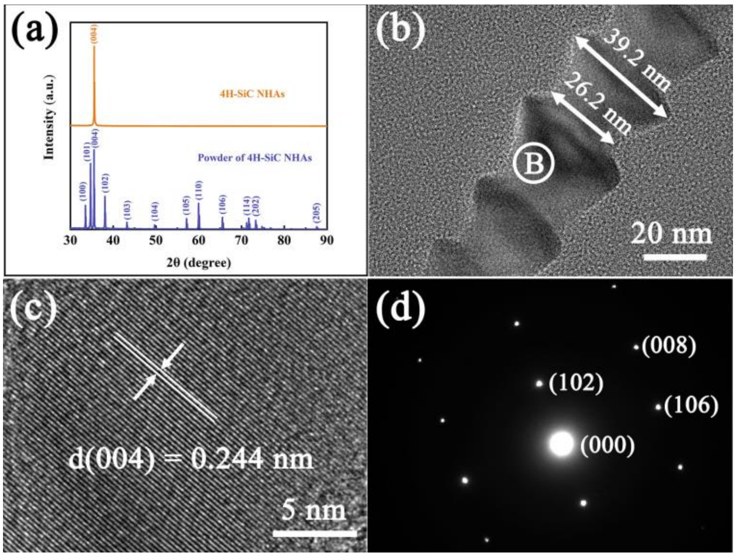

Fig. S2 (**a**) XRD results of NHAs and their powder. (**b**) TEM images of NHAs. (**c**) HRTEM image and (**d**) SAED patterns of the selected area B in (**b**)

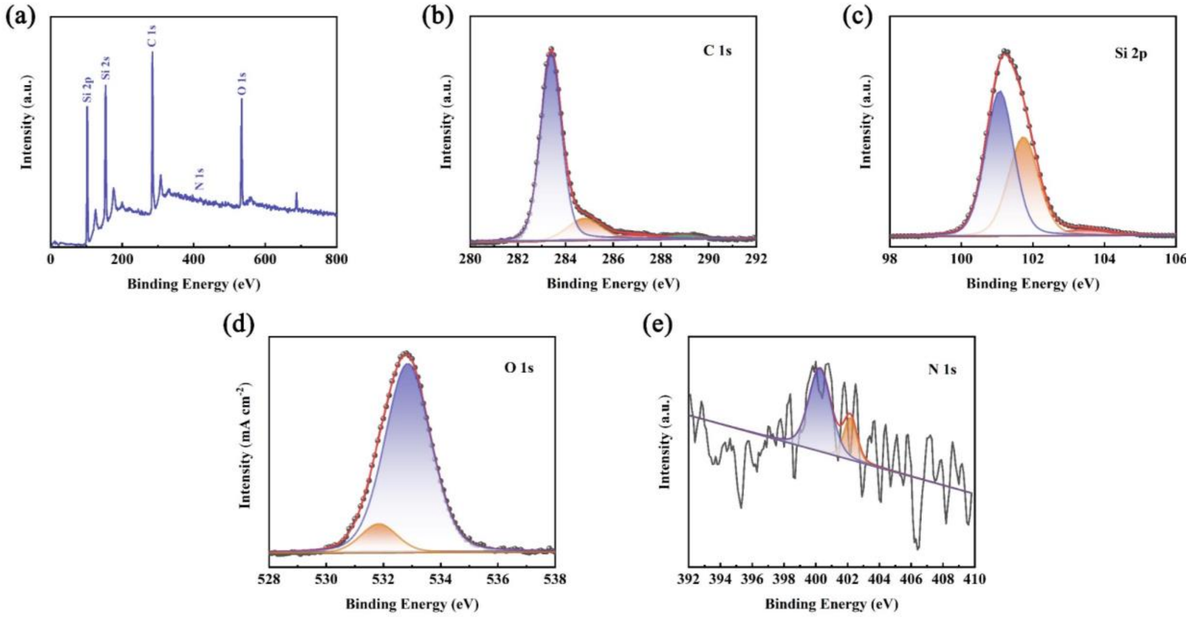

Fig. S3 (**a**) Full XPS spectrum of NHAs. Fine XPS spectrum of different elements: (**b**) C 1s; (**c**) Si 2p; (**d**) O 1s; (**e**) N 1s

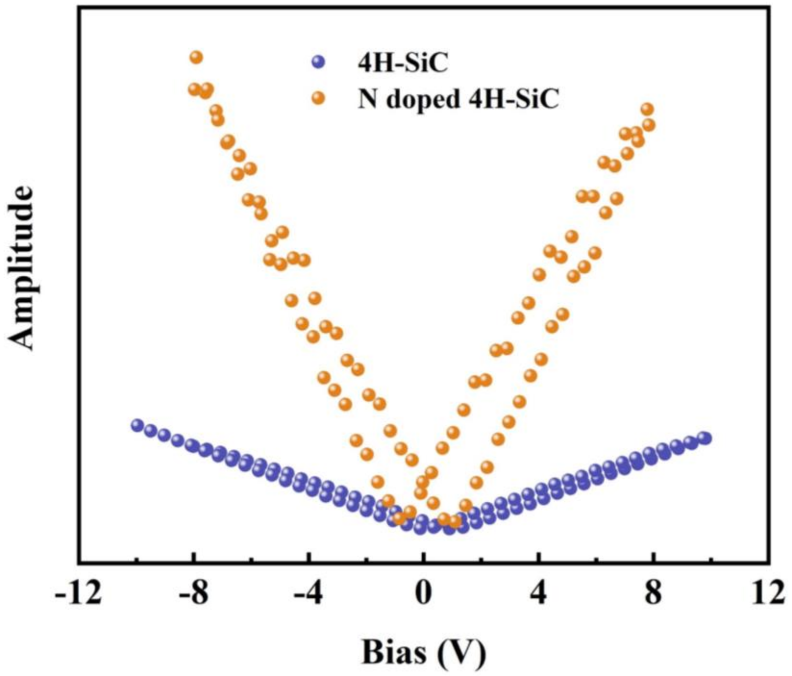

Fig. S4 PFM amplitude-voltage butterfly curve of 4H-SiC and N doped 4H-SiC

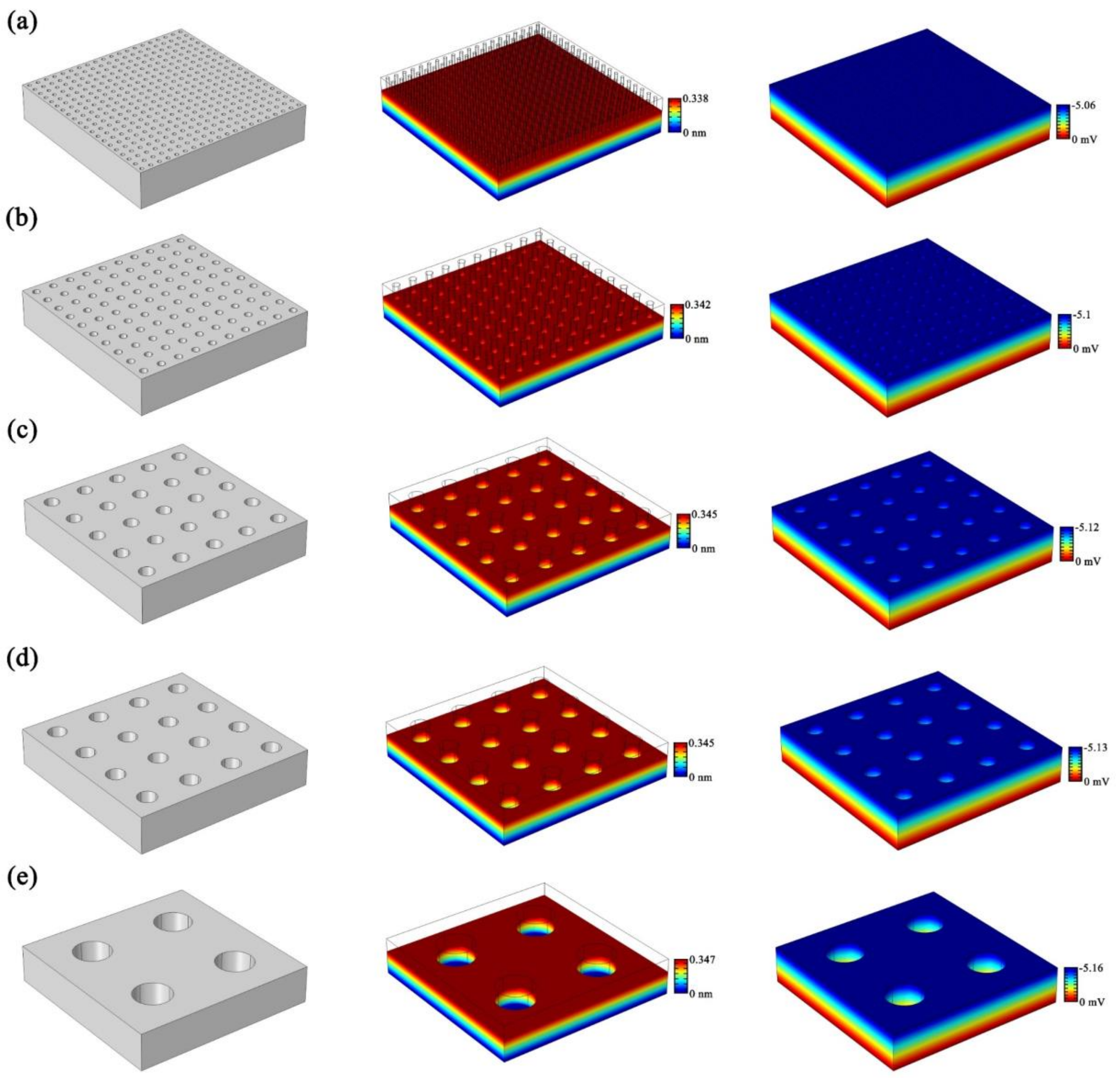

Fig. S5 Distribution of D and V of the units with different diameter: (**a**) 20 nm; (**b**) 40 nm; (**c**) 80 nm; (**d**) 100 nm; (**e**) 200 nm

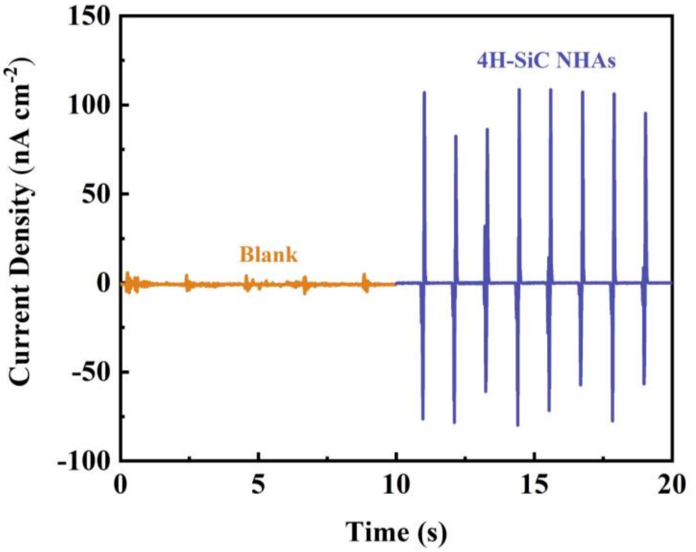

Fig. S6 Density of I_{sc} of PENG based on N doped 4H-SiC NHAs and the blank one

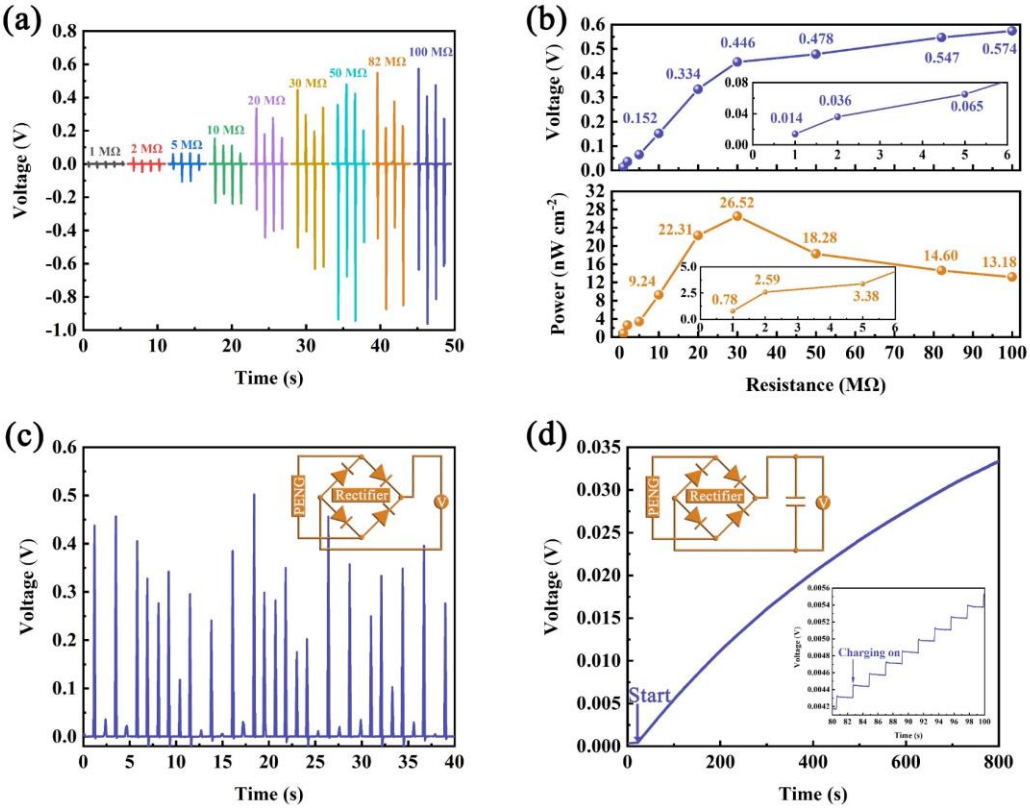

Fig. S7 (**a**) V_{oc} of PENG under resistive load varies from 1 MΩ to 100 MΩ. (**b**) The tendency of V_{oc} and power density of PENG under different resistances. (**c**) The voltage of PENG after bridge rectification. (**d**) The charging curve of 100 μF capacitor

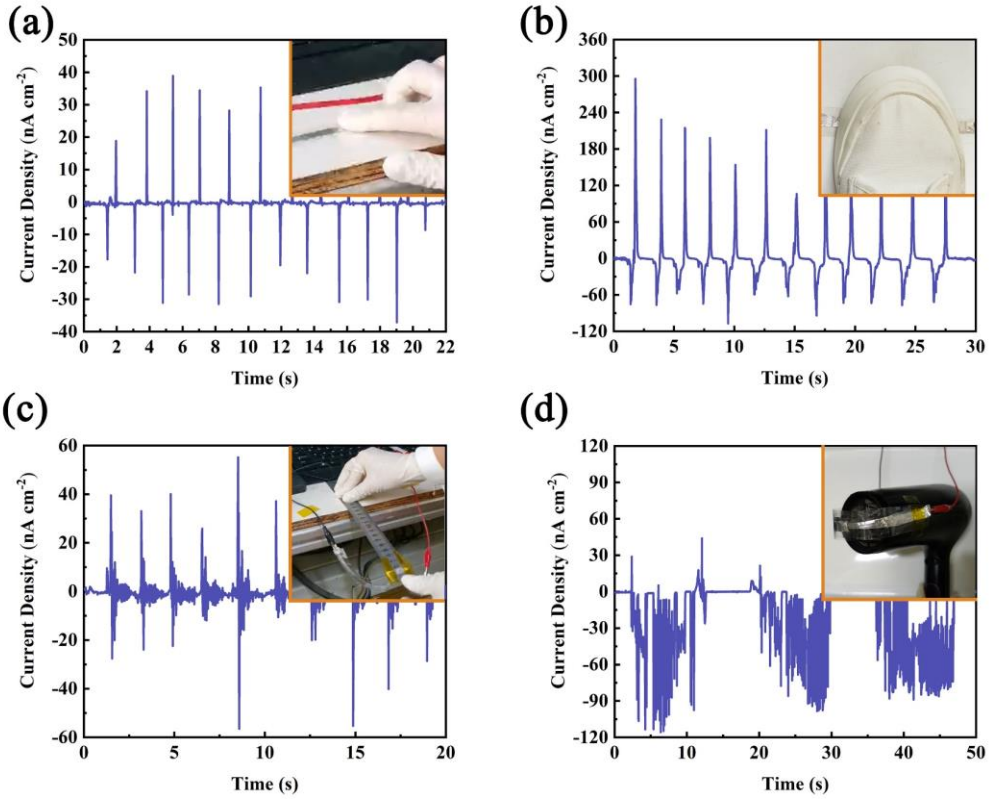

Fig. S8 Density of I_{sc} of PENG when stimulated in different ways: (**a**) finger tapping; (**b**) foot striking; (**c**) simulated cantilever beam; (**d**) simulated exhaust emission
